# Efficient Photocatalytic Degradation of Malachite Green and Cr(VI) Using Co-MOF and Bacterial Cellulose@Co-MOF Biocomposite: A Green Approach

**DOI:** 10.1021/acsomega.5c06750

**Published:** 2025-09-23

**Authors:** Krushika Mhalshekar, Subhesh Selvam, Aditya Sahoo, Mani Pujitha Illa, Mrunalini Gaydhane, Sharad Sontakke

**Affiliations:** † Nano Prakruti Research Laboratory, Department of Chemical Engineering, Birla Institute of Technology and Science, Pilani, K K Birla Goa Campus, Zuarinagar 403726, Goa, India; ‡ Battery Materials Laboratory, Department of Metallurgical and Materials Engineering, National Institute of Technology Tiruchirappalli, Tiruchirappalli 620015, Tamil Nadu, India

## Abstract

The persistent presence of synthetic dyes such as malachite green (MG) and inorganic heavy metals such as Cr­(VI) in industrial effluents poses a major environmental threat due to their toxicity and resistance to biodegradation. Their high solubility and bright colors make removal difficult, necessitating efficient and sustainable remediation techniques. Photocatalysis is an environmentally friendly water treatment technique that uses light energy to activate a photoactive catalyst, degrading pollutants into less harmful products, such as carbon dioxide and water. This study investigates the photocatalytic degradation of MG and Cr­(VI) using cobalt-based metal–organic frameworks (Co-MOF) and a bacterial cellulose-supported composite (BC@Co-MOF). The catalyst was synthesized using the precipitation method. The BC@Co-MOF composites were prepared using an *in situ* synthesis method. The as-synthesized materials were characterized by using a scanning electron microscope, energy dispersive X-ray spectrometry, powder X-ray diffractometer, photoluminescence spectroscopy, UV-diffuse reflectance spectroscopy, X-ray photoelectron spectroscopy, porosimetry, and thermogravimetric analysis. The effect of initial concentration of the target pollutant, solution pH, and source of light on the photocatalytic degradation was studied. The nanofibrous BC@Co-MOF composite enhanced the catalytically active sites, improved mass transfer, and facilitated efficient photodegradation. In the presence of UV radiation, BC@Co-MOF 6H demonstrated the highest 92.52% degradation of MG and 82.06% degradation of Cr­(VI) in 60 min under optimum conditions (10 ppm and neutral pH of the solution). The photocatalytic degradation of MG followed pseudo-first-order kinetics. The radical scavenger studies revealed the dominance of h^+^ radicals for the degradation of MG, whereas for the degradation of Cr­(VI), ^•^O_2_^–^, and h^+^ were the dominant reactive species. The underlying mechanism for the degradation of MG was proposed with the help of LC-MS analysis. The photocatalytic degradation of an aqueous solution containing a mixture of MG and Cr (VI), in the presence of BC@Co-MOF 6H and under solar light, showed 88.36 and 81.65% degradation of MG and Cr (VI), respectively. The degradation of the mixed pollutants revealed the synergistic effect of adsorption and photodegradation. The BC@Co-MOF 6H catalyst exhibited excellent photocatalytic activity when reused for up to 4 cycles. The BC@Co-MOF 6H catalyst also exhibited promising results for the treatment of real-time industrial effluent, indicating its practical applicability. Furthermore, phytotoxicity studies confirmed the safe nature of treated water, making BC@Co-MOF a green, recyclable, and highly effective photocatalyst for wastewater treatment.

## Graphical abstract

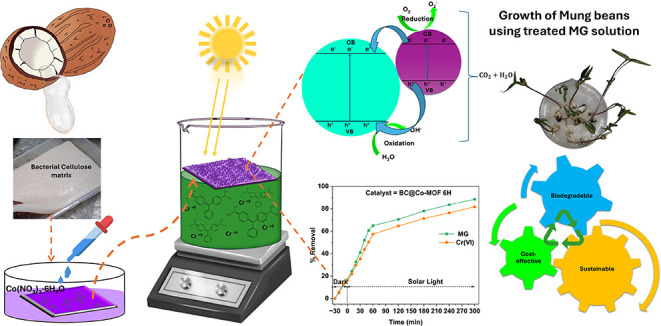

## 1 Introduction

Malachite green (MG), a cationic triphenylmethane dye, is widely used in the textile, leather, and paper industries.^1^ The triphenylmethane group dyes, such as MG, have intense color and excellent color fastness, retaining their brightness and resistance to fading over time during repeated washing cycles. Even at low concentrations, these dyes pollute water bodies and are considered major textile pollutant. When MG is discharged into water bodies, either untreated or treated partially, it causes ecological imbalance and poses serious threats to the environment and public health. Due to its bright color and high water solubility, MG lowers the amount of light passing through the water, disrupting the aquatic photosynthesis process and limiting the reproductive ability of aquatic organisms. Exposure to MG has been reported to cause severe effects on human health, such as allergies, dermatitis, and skin irritation. A long-term exposure to MG has been linked with its mutagenic and carcinogenic effects.^2,3^ This necessitates a strict regulatory threshold and efficient water treatment techniques for the removal of MG. Due to its cationic nature, MG exhibits strong interactions with anionic functional groups on substrates, thereby contributing to persistent adsorption and resistance to removal.^4^ Similarly, Cr­(VI), a highly toxic and carcinogenic heavy metal ion commonly found in industrial effluents from the electroplating, tanning, and pigment sectors, poses a serious environmental threat. Its high solubility and mobility enable it to exist in aquatic habitats, posing toxic and carcinogenic threats to the ecosystems and human health.^5,6^ Cr­(VI) inhibits enzymatic reactions causing oxidative stress in living organisms. According to WHO criteria, the acceptable limit of Cr­(VI) in drinking water is as low as 0.1 mg/L; therefore, the effective removal of this contaminant is crucial.^7,8^

Advanced oxidation processes, such as photocatalysis, have garnered significant attention as an environmentally friendly water treatment technique.^9^ Photocatalysis or photocatalytic degradation utilizes light energy to activate a catalyst and creates electron–hole (e^–^/h^+^) pairs on its surface. These charge carriers (e^–^/h^+^) participate in redox reactions, where the reduction of dissolved oxygen by the electrons produces superoxide radical (^•^O_2_^–^), and water oxidation leads to the formation of hydroxyl radicals (^•^OH).^10,11^ These reactive oxygen species are highly effective in degrading complex aromatic structures of MG and reducing Cr­(VI) to less toxic Cr­(III). These photocatalysis processes lead to the mineralization of pollutants into benign end-products such as carbon dioxide and water.^12^ For the practical application of photocatalysis, a photocatalyst that exhibits low electron–hole recombination rates and has a narrow band gap corresponding to the absorbance of visible light should be considered.

Metal organic frameworks (MOFs) represent a novel class of porous crystalline materials synthesized from metal ions or clusters coordinated with organic ligands through strong coordination bond. Its high surface area and well-defined pore structure facilitate uniform distribution of electro-active sites, promote rapid ion diffusion, and efficient mass transfer, making it attractive in various research areas.^13,14^ Among MOFs, Co-MOF, also known as ZIF-67, has demonstrated promising application in photocatalysis due to its unique properties. The catalytic activity of Co-MOF is significantly enhanced by the presence of redox-active Co centers (Co^2+^/Co^3+^), which facilitate the efficient oxidation–reduction reactions. The optical activity of cobalt renders Co-MOF photoactive under visible light; additionally, its abundant active sites contribute to its effectiveness as a photocatalyst in redox-driven degradation processes.^15^

The photocatalytic application of as-synthesized powdered MOFs faces limitations such as particle agglomeration, structural collapse, and loss of active catalytic sites during reuse. Their performance often declines over time due to poor mechanical stability and leaching of active components.^16^ This makes handling, recovery, and reuse difficult for practical applications. Films or fibers are preferred over powdered photocatalysts for water treatment due to their practical advantages in recovery, reusability, and environmental safety. They remain immobilized on a substrate, allowing easy handling and reuse across multiple cycles.^17,18^ Fibers reduce the nanoparticle leaching risk and are compatible with continuous-flow systems. They offer better light penetration and stability, making them an efficient, sustainable, and scalable approach for real-world wastewater treatment. To circumvent the disadvantages associated with the use of powdered MOFs and enhance the structural stability of the photocatalyst, in this work, we report a strategy of employing Bacterial Cellulose (BC) as a biotemplate. BC is an eco-friendly, renewable nanofibrous substrate characterized by its highly porous structure and provides a strong mechanical support for the dispersion of Co-MOF on its surface and additional adsorption sites for the uptake of the target pollutants.

In the present study, a BC@Co-MOF was synthesized via *in situ* growth of Co-MOF on BC, with the aim of enhancing the surface functionality and recyclability. The as-synthesized materials were characterized using scanning electron microscopy (SEM), energy dispersive X-ray spectrometry (EDS), powder X-ray diffractometer (XRD), photoluminescence (PL) spectroscopy, UV-diffuse reflectance spectroscopy (UV-DRS), X-ray photoelectron spectroscopy (XPS), porosimetry, and thermogravimetric analysis (TGA). Photocatalytic degradation of MG and Cr­(VI) was investigated in the presence of Co-MOF and BC@Co-MOF. The effect of the initial concentration of the target pollutant, solution pH, and source of light on the photocatalytic degradation was studied. Radical scavenging experiments were performed to identify the primary reactive species involved in the degradation. The experimental results were fitted to describe suitable degradation kinetics. The underlying degradation mechanism is proposed with the help of LC-MS analysis. Recycle studies were performed to test the reusability of the synthesized composite material. A photocatalytic degradation of real-time pharmaceutical industry effluent was performed to demonstrate the practical application of the present work. To confirm the environmental safety of the treated water, phytotoxicity studies were performed using chickpea (*Cicer arietinum* L.) and mung bean (*Vigna radiata* L.) seeds. This work demonstrates the potential of BC@Co-MOF as an efficient, recyclable, and solar-active photocatalyst for the sustainable treatment of dye- and metal-contaminated wastewater.

## 2 Materials and Methodology

### 2.1 Materials

Cobalt nitrate hexahydrate (Co­(NO_3_)_2_·6H_2_O, 99.5% pure, Sisco Research Laboratories Pvt. Ltd., India) was used as precursor, and 2-methylimidazole (99.5% pure, Sisco Research Laboratories Pvt. Ltd., India) was used as a ligand for the synthesis of Co-MOF. MG dye (Loba Chemie, India) and potassium dichromate (99% pure, Merck Life Sciences Pvt. Ltd., India) were used as target pollutants in the photocatalytic degradation studies. To adjust the pH of the solution, HCl (36.5% pure, Thermo Fischer Scientific India Pvt. Ltd.) and NaOH (99.7% pure, Thermo Fischer Scientific India Pvt Ltd.) were used. To detect Cr­(VI) from the solution, H_2_SO_4_ (98% pure, Merck Life Sciences Pvt. Ltd., India) and Diphenyl carbazide (Loba Chemie, India) were used. For the radical scavenger studies, tert-butanol (TBA, 99.7% pure, Sigma-Aldrich), benzoquinone (BQ, 98% pure, Sigma-Aldrich), and ammonium oxalate (AO, 99% pure, Loba Chemie, India) were used as scavenging agents. Deionized (DI) water (resistivity 18.2 MΩ·cm, Avidity Science) was used in all studies. All of the chemicals were of analytical grade and used as supplied, without any purification.

### 2.2 Synthesis of Co-MOF

To synthesize Co-MOF, an 80 mM aqueous solution of cobalt nitrate hexahydrate [Co­(NO_3_)_2_·6H_2_O] was first prepared. Subsequently, a 0.4 M aqueous solution of 2-methylimidazole was added to the cobalt solution under ambient conditions. The mixed solution was allowed to precipitate without stirring for up to 12 h for the formation of Co-MOF crystals. The precipitated product was collected by centrifugation, washed multiple times with deionized water to remove unreacted precursors, and dried in a hot-air oven at 60 °C for 12 h. The resulting powder was stored as Co-MOF.

### 2.3 Pretreatment of Bacterial Cellulose (BC) Pellicle

BC was procured from the Kasturi coconut processing unit in Bangalore, where BC was produced through coconut water fermentation. The as-received BC was soaked in 1 M NaOH solution for 12 h, boiled at 100 °C to remove the bacterial cell debris, and then washed with DI water until the pH reached 7. The treated BC samples were stored for further use.

### 2.4 Preparation of BC@Co-MOF Composites

An 80 mM aqueous solution of cobalt nitrate hexahydrate [Co­(NO_3_)_2_·6H_2_O] was prepared, and the pretreated BC samples were immersed in the solution and soaked for 24 h to facilitate cobalt ion absorption. After 24 h, a 0.4 M solution of 2-methylimidazole was added directly to the soaked BC, and the reaction was allowed to proceed. Samples were sequentially removed from the solution at different time intervals, namely, 1 hour (1H), 6 hours (6H), and 12 hours (12H). Each sample was washed with DI water to remove unreacted precursors and dried in a hot-air oven at 60 °C for 12 h. These samples were labeled as dried BC@Co-MOF composites. The formation of BC@Co-MOF was evident from the color change of the bacterial cellulose (BC) to purple upon the addition of the 2-methylimidazole, indicating the successful *in situ* growth of Co-MOF crystals on the BC biotemplate. The samples removed at different time intervals, namely 1 hour, 6 hour and 12 hour were labeled as BC@Co-MOF 1H, BC@Co-MOF 6H, and BC@Co-MOF 12H, respectively.

### 2.5 Characterization Techniques

The surface morphology and elemental composition of the synthesized Co-MOF and BC@Co-MOF composites were analyzed using field emission scanning electron microscopy (FESEM) (Quanta FEG 250 SEM, FEI) and energy dispersive spectroscopy (EDS), respectively. The X-ray diffraction (XRD) patterns of the prepared materials were recorded by using an X-ray diffractometer (Bruker-D8 Advance, Germany) equipped with Cu-Kα radiation. The XRD patterns were obtained at a step of 0.02°/min at room temperature in the 2θ range of 5–80°. The photoluminescence (PL) spectra of the synthesized materials were obtained using a 325 nm He–Cd laser 25 mW (LAB RAM HR Horiba, France). The band gap energy of the synthesized materials was calculated using absorbance and reflectance data from UV–visible diffuse reflectance spectroscopy (UV–vis DRS, UV-2600, Shimadzu, Japan). The electronic states of the synthesized material were examined by using X-ray photoelectron spectroscopy (XPS, Thermo Scientific K-Alpha). The surface area of the synthesized materials was measured by the Brunauer–Emmett–Teller (BET) method using a 3-Flex instrument (Micromeritics; 3-Flex). The decomposition behavior, thermal stability, and robustness of the synthesized materials were studied using thermogravimetric analysis (TGA) (Discovery SDT 650, TA Instruments) in the temperature range of 25–600 °C and at a 10 °C/min heating rate.

### 2.6 Photocatalytic Degradation Studies

The photocatalytic degradation experiments under UV light were conducted by using a jacketed quartz photoreactor. In this, an arc filament of a 125 W high-pressure mercury vapor lamp (HPLN, Philips, India) emitting predominantly at 365 nm was placed inside the quartz tube by carefully removing the outer cover of the lamp. Cold water was continuously circulated through the quartz tube’s jacket with the help of a submersible pump. The photocatalytic degradation experiments in the presence of visible light were performed in natural sunlight at atmospheric conditions in the month of March between 10.30 am to 3.30 pm (IST).^19^ In a typical experiment, 100 mL of aqueous solution of the target pollutant with a known concentration containing 50 mg of the desired catalyst was placed over a magnetic stirrer at a distance of 5 cm from the UV light source. For the above experimental conditions, the light intensity recorded at the surface of the solution under UV light was 8000 lx, and under direct sunlight was 69,700 lx. To study the adsorption–desorption equilibrium, the solution was first stirred in the dark for 30 min. The reaction mixture was then exposed to a light source to study the photocatalytic degradation. Samples were collected from the reaction mixture at periodic intervals and centrifuged. Following the catalyst separation process, the absorbance of the solution was recorded at λ_max_ = 615 nm for MG using a UV–vis spectrophotometer (K Lab Optizen Alpha, Korea) to quantify the photocatalytic activity. The amount of Cr­(VI) in the solution was estimated using the 1,5-diphenyl carbazide (DPC) method. In this, the aliquots (2 mL) of the Cr­(VI) solution were mixed with 1 mL of H_2_SO_4_ and 1 mL of freshly prepared 0.2 g of 5 g/L DPC in acetone.^20^ The absorbance peak of the resulting reddish violet complex was recorded at λ_max_ = 540 nm. The % degradation of the target pollutant was calculated using the following:

1 $$\%\;De\mathrm{grad}ation=\frac{C_{0}-C}{C_{0}}\times 100$$

where, *C*_0_ is the initial concentration of the pollutant, and *C* is the concentration after irradiation time, *t*. The effect of the initial concentration of the target pollutant and solution pH on the photocatalytic degradation was studied. The identification of the primary reactive species involved in degradation was performed by radical scavenging experiments. The *tert*-Butyl alcohol (TBA), benzoquinone (BQ), and ammonium oxalate (AO) were used as scavenging agents for ^•^OH, ^•^O_2_^–^, and h^+^, respectively. In this, 2.5 mM of the scavenging agent was added to 100 mL of an aqueous solution containing the target pollutant at an initial concentration of 10 ppm. The above solution was subjected to photocatalytic degradation using BC@Co-MOF 6H under UV or solar light. To check the efficiency of the composite catalyst (BC@Co-MOF), a mixture of MG dye and Cr­(VI) was subjected to photocatalytic degradation under UV and solar light.

To study the reusability of the photocatalyst, it was recovered from the solution, washed thoroughly with water and ethanol, and heated in a hot air oven for 1 h at 100 °C. The dried material was then reused in a fresh photocatalytic degradation experiment. The process was repeated for up to four cycles. A liquid chromatograph coupled with a mass spectrometer (LC-MS, model: 6460C Triple Quadrupole, Agilent Technologies) with a positive electrospray ionization (ESI) source was used to investigate the intermediate formed during the photocatalytic degradation process. The LC-MS was fitted with a Zorbax SB-C18 column (2.1 × 50 mm^2^, pore size of 1.8 μm). The mobile phase consisted of water (solvent A) and acetonitrile (solvent B) at a flow rate of 0.2 mL/min. The mass range was 100–700 *m*/*z*. To demonstrate the real-world application of the synthesized material, photocatalytic degradation of real-time pharmaceutical industry effluent was performed in the presence of BC@Co-MOF 6H.

### 2.7 Phytotoxicity Studies

The germination of chickpea (*Cicer arietinum* L.) and mung bean (*Vigna radiata* L.) seeds was investigated in order to assess the phytotoxicity of photocatalytically treated dye solutions. In this, photocatalytic degradation of 10 ppm dye solution was performed under UV light using various materials, namely, as-prepared BC, Co-MOF, and BC@Co-MOF 6H. The seeds were presoaked in the treated dye solution remaining at the end of each of the above degradation experiment. The seeds were then placed over wet cotton in Petri dishes and incubated for 24 h in the dark at 25 ± 2 °C for germination. The germinated seeds were transferred to Petri dishes containing small amounts of distilled water (control), treated dye solution, and an untreated dye solution. The Petri dishes were prepared in triplicate and kept in the dark inside an incubator at 25 ± 2 °C for 4 days.

The length of the grown shoots was measured, and the % growth inhibition was calculated using eq 2:

2 $$\%\;Growth\;Inhibition={\frac{length\;of\;control-length\;of\;test}{length\;of\;control}}^{\;}\times 100$$

The above-mentioned experimental protocol was adopted from Kurian et al.^21^

## 3 Results and Discussion

### 3.1 Characterization of the Prepared Materials

The FESEM micrographs of the Co-MOF and BC@Co-MOF are shown in Figure 1. The Co-MOF exhibited a cluster of particles that resembled a densely packed, plate-like crystal with uneven edges (Figure 1a).^22^ The BC appeared to be densely packed fibers with randomly oriented morphology (Figure S1). The BC@Co-MOF 1H material showed rod-like Co-MOF on the fibrous BC (Figure 1b). The BC@Co-MOF 6H material showed a transient stage of Co-MOF grown on the BC matrix (Figure 1c). The rod-like structures of Co-MOF in this case (BC@Co-MOF 6H) were longer in length and uniformly distributed over the BC matrix, compared to the BC@Co-MOF 1H material. Figure 1d shows the FESEM image of BC@Co-MOF 12H depicting its interconnected, elongated, and thicker plate-like structure covering the BC matrix more extensively. The surface of BC@Co-MOF 12H appeared densely coated with Co-MOF, indicating a significant *in situ* growth of Co-MOF on the BC matrix after 12 h immersion.

**Figure 1:**
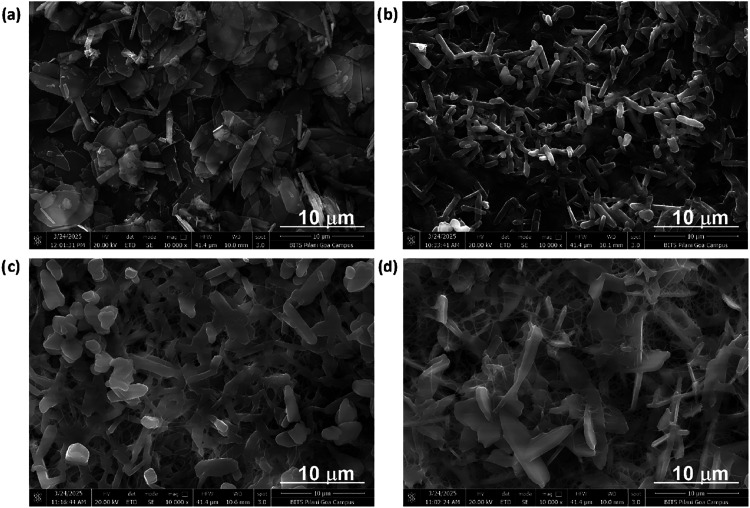
FESEM images of (a) Co-MOF, (b) BC@Co-MOF 1H, (c) BC@Co-MOF 6H, and (d) BC@Co-MOF 12H.

The EDS spectra of Co-MOF and BC@Co-MOF composites are presented in Figure 2. Carbon (C), Nitrogen (N), Oxygen (O), and Cobalt (Co) were identified as the main elements that match the elemental composition of the organic ligands, the cobalt centers of the MOF, and the cellulose structure. The effective synthesis was confirmed by the considerable presence of Co, C, N, and O in Co-MOF. The EDS analysis of BC@Co-MOF 1H exhibited an increase in C and O content due to the inclusion of bacterial cellulose matrix in the composite. With Co, C, N, and O more evenly distributed, BC@Co-MOF 6H demonstrated enhanced Co-MOF loading on the cellulose structure while retaining an adequate N content. The EDS analysis of BC@Co-MOF 12H exhibited the highest (34 wt %) cobalt concentration among all samples, indicating significant Co-MOF deposition over the bacterial cellulose surface due to the extended immersion. The EDS spectra confirmed the successful incorporation of Co-MOF onto BC in all of the composite materials. The increase in Co content in materials that were immersed for longer durations can be attributed to the greater nucleation and growth of Co-MOF crystals on the BC matrix over an extended immersion time.

**Figure 2:**
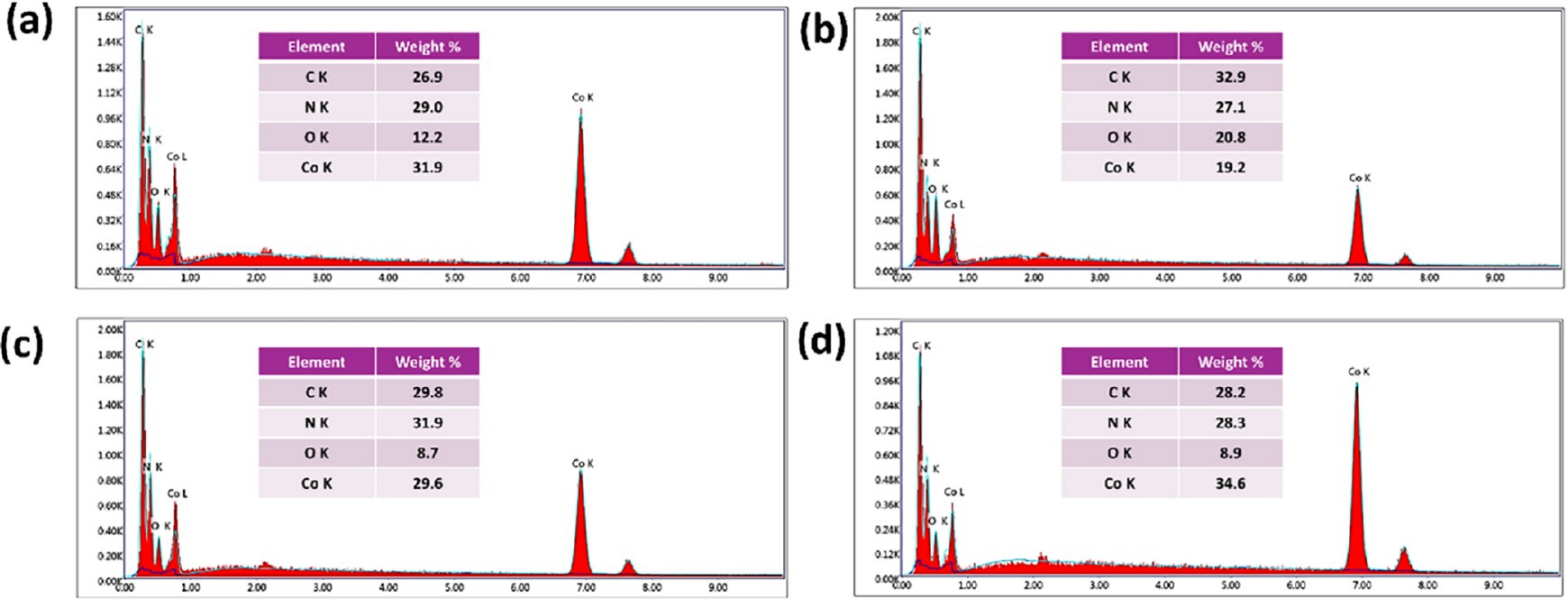
EDS spectra of (a) Co-MOF, (b) BC@Co-MOF 1H, (c) BC@Co-MOF 6H, and (d) BC@Co-MOF 12H.

From the XRD patterns depicted in Figure 3a, it was observed that pure Co-MOF and its composites with BC showed notable variations in the crystallinity and structural integrity. In the case of Co-MOF, sharp diffraction peaks are visible, indicating the highly crystalline nature of the material. The peaks at 2θ values of 7.3, 12.98, 17.24, 18.04, 24.9, 29.64, and 36.31° correspond (100), (002), (202), (013), (113), (223), and (151) crystal planes of Co-MOF, respectively.^23–27^ The diffraction peaks of Co-MOF are in agreement with the simulated pattern of ZIF-67 (Figure 3b) obtained by using Materials Studio software. The XRD pattern of BC shows the presence of distinct peaks at the 2θ values of 14.45, 16.72, and 22.77° corresponding to the (100), (010), and (110) crystal planes, respectively.^28,29^ After incorporating Co-MOF on BC, in BC@Co-MOF composites, most of the characteristic peaks of Co-MOF are retained with a slight decrease in intensity and peak broadening. The presence of intense diffraction peaks corresponding to Co-MOF and the absence of peaks corresponding to BC in the BC@Co-MOF 1H suggests *in situ* nucleation and growth of crystalline MOF structures on the surface of BC. For BC@Co-MOF 6H, the intensity of the peak at 2θ = 17.75° is significantly increased due to the orientation or development of larger crystallites of the Co-MOF.^30^ The pronounced diffraction peaks of the composite material are more aligned toward Co-MOF, which shows improved MOF growth on BC and enhanced crystallinity. With the increase in the immersion time, the diffraction peaks appeared sharper and more intense, indicating superior crystallinity.

**Figure 3:**
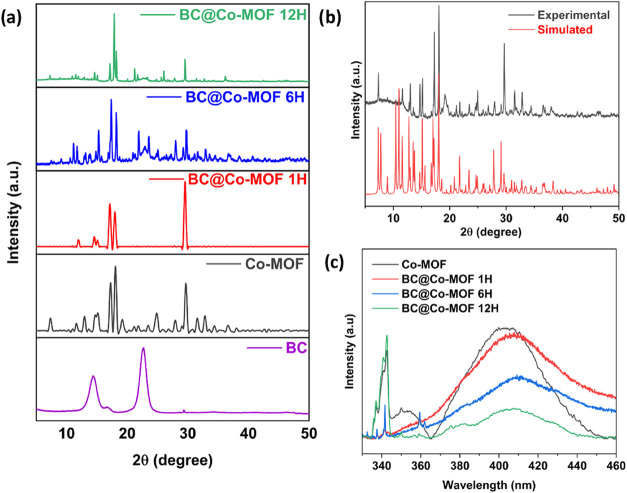
(a) XRD pattern of BC, Co-MOF, and their composites, (b) simulated XRD pattern of Co-MOF, and (c) PL spectra of Co-MOF and its composites on BC.

The separation of the photogenerated electron–hole pair plays a critical role in determining the photocatalytic efficiency. Photoluminescence (PL) spectroscopy was used to evaluate the recombination rates of the charge carriers. The higher PL intensity typically reflects faster recombination, while a reduced intensity indicates more efficient charge separation and longer carrier lifetimes. Figure 3c depicts the PL spectra of the Co-MOF and its composites. Co-MOF exhibited the highest emission intensity, indicating a higher recombination rate of charge carriers. The PL intensity of all of the BC@CO-MOF composites was lower compared to Co-MOF, suggesting effective charge separation of these materials due to the integration of Co-MOF on the BC matrix. Among the composite materials, lowest PL intensity was observed for BC@Co-MOF 12H.^31,32^

The optical band-gaps of the prepared materials was estimated with the help of UV–vis DRS analysis. Figure 4 shows the UV-DRS spectra and Tauc plots of the Co-MOF and BC@Co-MOF composites. By using Kubelka–Munk function, the band gap energies for Co-MOF, BC@Co-MOF 1H, BC@Co-MOF 6H, and BC@Co-MOF 12H were calculated as 3.29, 3.26, 3.14, and 3.20 eV, respectively. The pristine Co-MOF showed strong absorption in the UV region. The BC@Co-MOF 1H material showed a slight redshift, suggesting enhanced light harvesting due to improved dispersion and interfacial contact between the MOF and BC. BC@Co-MOF 6H showed the most significant band gap reduction, indicating optimal synergy between the Co-MOF and BC matrix. This enhancement can be attributed to the formation of interface-induced defect states, allowing more efficient light absorption in the visible region and promoting better electron–hole separation. BC@Co-MOF 12H showed a slight increase in the band gap, which can be attributed to excessive MOF agglomeration or surface saturation. Among the prepared materials, the BC@Co-MOF 6H composite displayed superior optical properties for visible-light-driven photocatalytic applications.

**Figure 4:**
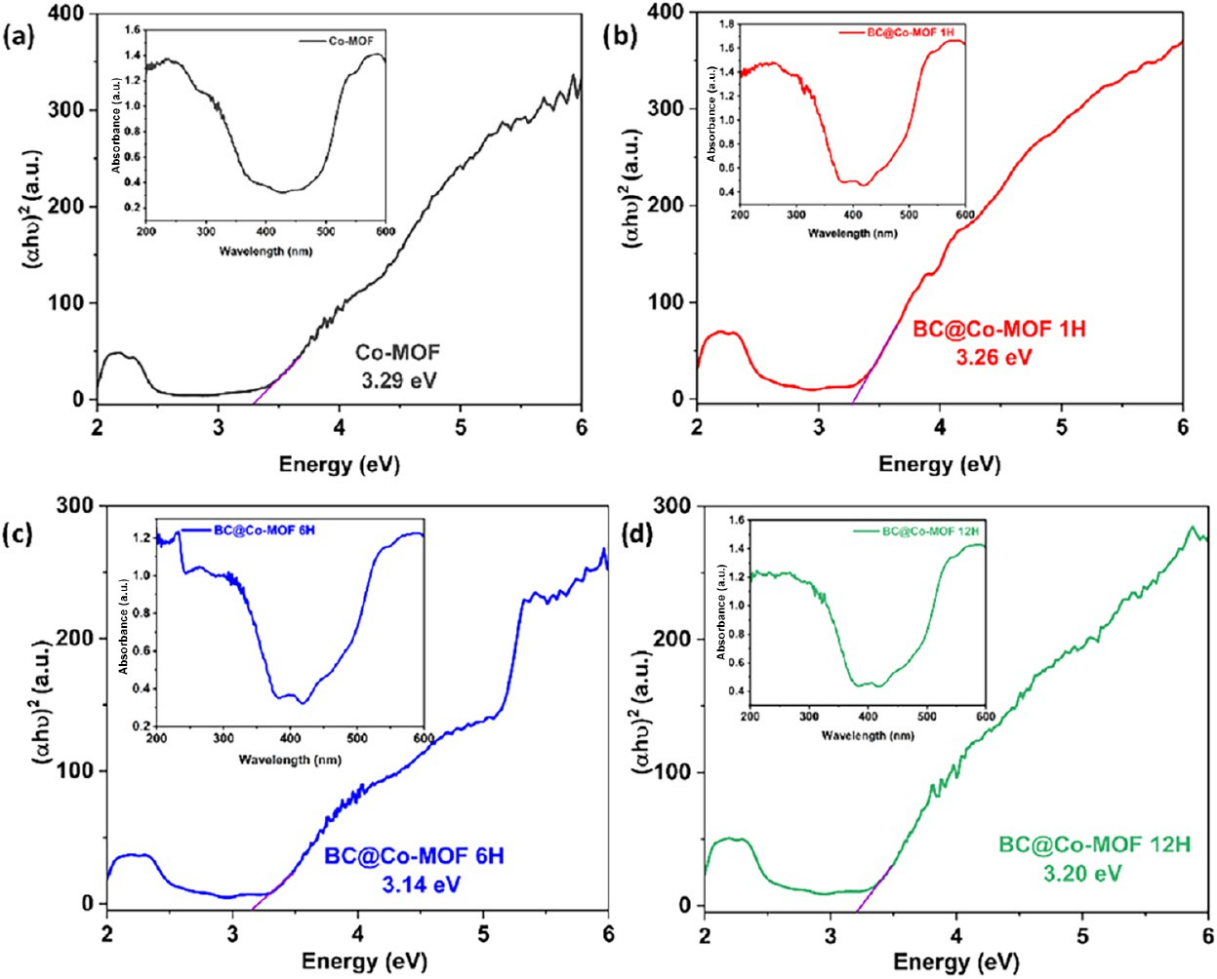
UV-DRS spectra and Tauc plot of (a) Co-MOF, (b) BC@Co-MOF 1H, (c) BC@Co-MOF 6H, and (d) BC@Co-MOF 12H.

The XPS spectra of the Co-MOF and BC@Co-MOF composite materials are shown in Figure S2. The core level XPS spectrum showed the presence of photoelectron peaks corresponding to C 1s (284.62 eV), N 1s (398.06 eV), O 1s (532.62 eV), and Co 2p (782.19 eV) in all of the materials. These results were in agreement with the elemental analysis performed using EDS. The presence of Co 2p photoelectron peaks in all of the composite materials provides evidence of the successful incorporation of Co in the composite materials. The high-resolution deconvoluted XPS spectra of Co 2p, C 1s, O 1s, and N 1s in the BC@Co-MOF 6H are shown in Figure 5. In Figure 5a, the dominant Co 2p_3/2_ and 2 p_1/2_ peaks can be observed at binding energies of 781.05 and 796.75 eV, arising from Co^3+^. The notable satellite peaks appearing on the higher binding energies (785.81 and 802.53 eV) indicate the presence of Co^2+^. The peaks at binding energies of 284.7, 285.5, and 286.7 eV in the high-resolution spectrum of C 1s (Figure 5b) were associated with C–C/C=C, C=N, and C–N appearing from the BC and imidazole linker. The pronounced peaks appearing at the binding energy of 530.88 eV in the high-resolution spectrum of O 1s and 399.68 eV in the high-resolution spectrum of N 1s depict the presence of Co–O and Co–N linkages, respectively, which indicates a strong interaction between Co-MOF and BC in the composite material.^33,34^

**Figure 5:**
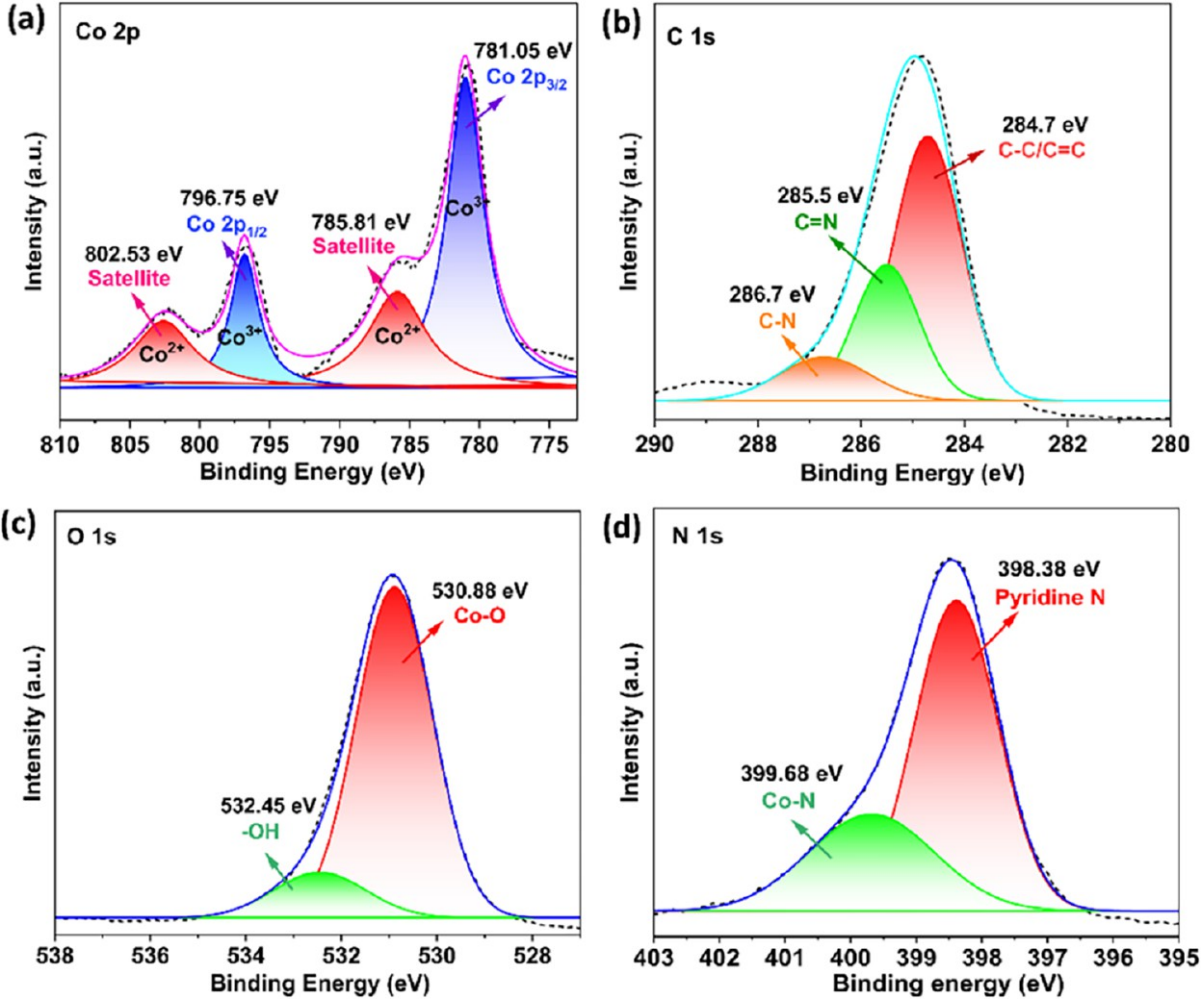
High-resolution deconvoluted spectrum of (a) Co 2p, (b) C 1s, (c) O 1s, and (d) N 1s in the BC@Co-MOF 6H.

The surface areas of synthesized Co-MOF and its composites are summarized in Table 1. The surface area of Co-MOF was higher compared to the composite material. The surface area for the composite materials followed the following order: BC@Co-MOF 1H > BC@Co-MOF 6H > BC@Co-MOF 12H. The decrease in surface area with an increase in immersion time for the composite material can be attributed to the presence of larger-sized particles of Co-MOF on the surface of BC during the *in situ* growth process.

**Table 1:** BET Surface Area of Co-MOF, BC@Co-MOF 1 H, BC@Co-MOF 6H, and BC@Co-MOF 12H

sr. no.	catalyst	surface area (m^2^/g)	pore volume (cm^3^/g)	average pore diameter (nm)
1	Co-MOF	164.08	0.1681	4.81
2	BC@Co-MOF 1H	31.80	0.04167	5.8
3	BC@Co-MOF 6H	4.4	0.008654	19.20
4	BC@Co-MOF 12H	2.9	0.002966	11.35

Figure 6 shows the TGA plots of the Co-MOF and BC@Co-MOF composites. The Co-MOF exhibited good thermal stability without any significant weight loss up to 270 °C. However, above 270 °C, a significant weight loss was observed, indicating thermal decomposition of the material.

**Figure 6:**
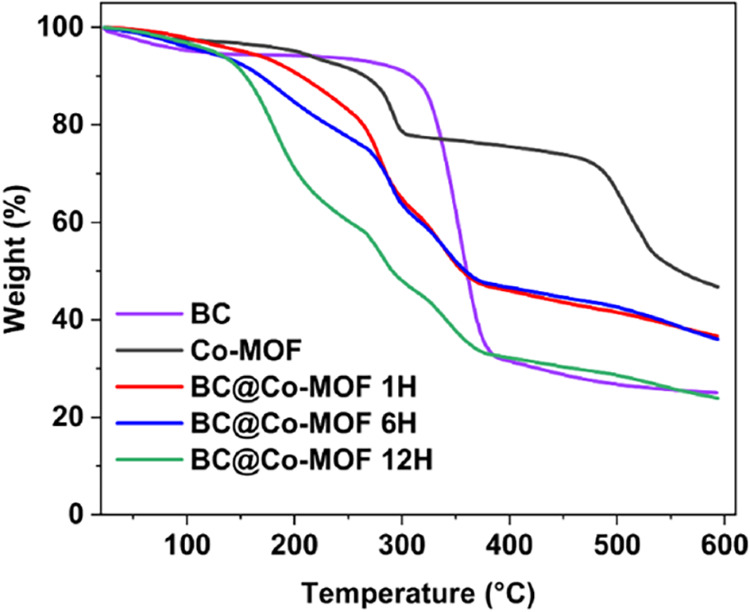
TGA plots of Co-MOF and its composites on BC.

The pure BC was thermally stable up to 250 °C; however, it underwent a major weight loss between 250 and 370 °C due to thermal degradation of the cellulose backbone.^35^ The TGA of composite materials showed a decrease in thermal stability compared to Co-MOF and BC. As the temperature increased, there was a significant weight loss of the composite materials due to the degradation of the organic linker and the breakdown of the polysaccharide chain within the bacterial cellulose. Among the composites, BC@Co-MOF 12H recorded the highest weight loss, suggesting that the longer synthesis time results in higher overgrowth and defects, weakening the resistance toward thermal decomposition.^34^

### 3.2 Photocatalysis Studies

#### 3.2.1 Effect of Initial Concentration of the Target Pollutant

The effect of the initial concentration on the photocatalytic degradation of MG and Cr­(VI) was studied using Co-MOF and BC@Co-MOF composites under UV light. The effect of initial concentration on the photocatalytic degradation of MG was studied in the range of 10–100 ppm. The results of the above experiments are shown in Figure 7. The results of the photocatalytic degradation studies were also compared with the control experiment under the same experimental conditions but in the absence of any catalyst (no catalyst or photolysis). The photocatalytic degradation of MG using any of the synthesized catalysts was superior compared with the photolysis experiment. At a low initial concentration of 10 ppm, the % MG degradation using Co-MOF, BC@Co-MOF 1H, BC@Co-MOF 6H, and BC@Co-MOF 12H was 86.82, 66.22, 91.81, and 80.40%, respectively. Increasing the initial concentration resulted in lower % MG degradation using the same catalyst. With an initial concentration of 50 ppm (Figure 7b) and 100 ppm (Figure 7c), the % MG degradation using Co-MOF was 42.06 and 35.41%, respectively. Among the synthesized materials, BC@Co-MOF 6H displayed the highest photocatalytic activity for the degradation of MG. The superior photocatalytic activity of the BC@Co-MOF 6H can be attributed to its narrower band gap energy, moderate crystallinity, and superior charge-separation properties. The decrease in % MG degradation with an increase in the initial concentration under the same catalyst can be attributed to the screening effect, in which excess dye molecules absorb the incident light and prevent it from interacting with the catalyst.^36,37^ The observed results are consistent with previous literature on photocatalytic dye degradation. Kavitha et al. observed a decrease in the photocatalytic degradation of MG when the initial concentration of the dye was increased. The authors attributed this phenomenon to the reduction in the surface active sites of the catalysts and inhibition of the light penetration at high initial concentration of the MG dye solution.^38^ Another study by Bilal et al. observed a decrease in the photodegradation efficiency with an increase in the initial concentration of Congo Red dye using a ZF@CM photocatalyst. Generally, it is proposed that the presence of more dye molecules at the higher concentration lowers the visible light penetration and thereby the generation of reactive oxygen species (ROS) in the photocatalysis process.^39^

**Figure 7:**
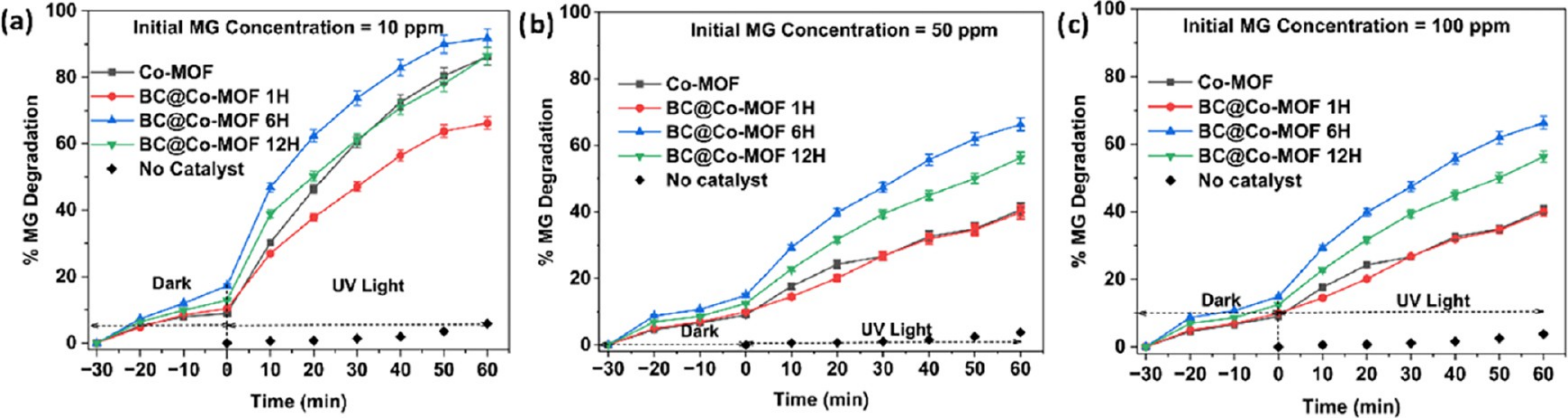
Effect of the initial concentration on the photocatalytic degradation of MG (a) 10 ppm, (b) 50 ppm, and (c) 100 ppm.

A similar trend was observed for the photocatalytic degradation of Cr­(VI), where the increase in the initial concentration decreased the % Cr­(VI) degradation. Figure 8 shows the photocatalytic degradation of MG dye and Cr­(VI) using the BC@Co-MOF 6H catalyst at different initial concentrations. At lower initial concentrations, the presence of sufficient active sites enables the photocatalyst to efficiently absorb and reduce Cr­(VI) ions. However, at higher concentrations, active sites become saturated, leading to incomplete interactions between the catalyst and Cr­(VI) ions.^40^

**Figure 8:**
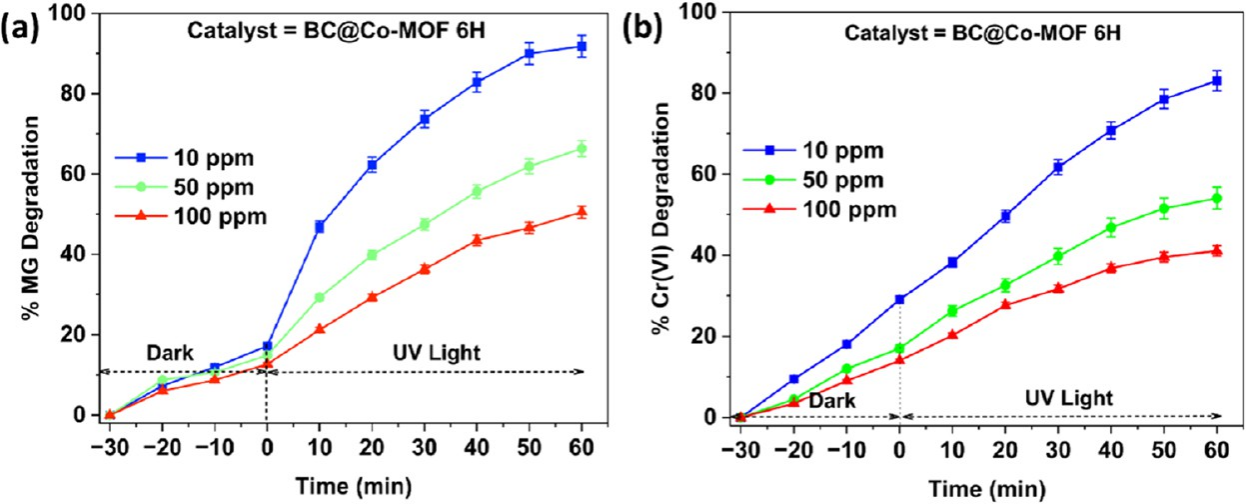
Photocatalytic performance of BC@Co-MOF 6H for the degradation of (a) MG and (b) Cr­(VI) at different initial concentrations.

For comparison purposes, a summary of various treatment methods, including adsorption, oxidation, and photocatalysis, reported for the removal of MG dye and Cr­(VI) has been compiled in Tables S1 and S2.

#### 3.2.2 Effect of pH

The photocatalytic degradation efficiency of BC@Co-MOF 6H was evaluated by varying the pH of the reaction solution as acidic (pH < 3), neutral (pH ≈ 7), and alkaline (pH > 10). The above experiments were performed under UV light with an initial concentration of 10 ppm, and the results are shown in Figure 9a,b for MG and Cr­(VI), respectively. For the degradation of MG, basic solution pH (pH > 10) favored the process, and approximately 95% degradation was observed within 60 min. The % MG degradation under neutral pH conditions was about 90%. However, when the solution pH was acidic (pH < 3), there was only 50% MG degradation. In acidic environments, protonation causes a catalyst’s surface to become positively charged, repelling cationic MG dye molecules and lowering degradation efficiency. Further, the low availability of hydroxide ions (OH^–^) in acidic pH conditions hinders the generation of highly reactive hydroxyl radicals (^•^OH), reducing the photocatalytic activity. On the other hand, the basic pH conditions favor the electrostatic attraction between the catalyst surface and dye molecules which results in higher degradation. Further, the maximum degradation at basic and neutral pH conditions can be attributed to the availability of optimum reactive oxygen species to target the degradation of the dye molecules.^40,41^ The effect of pH on the photocatalytic degradation of Cr­(VI) showed 88, 82, and 75% degradation of Cr­(VI) under acidic, neutral, and basic conditions, respectively. The photocatalytic degradation of Cr­(V) was favorable under acidic pH conditions. This is due to the electrostatic attraction between the negatively charged CrO_4_^2–^ and Cr_2_O_7_^2–^ ions and the catalyst surface under the acidic pH conditions.^42^

**Figure 9:**
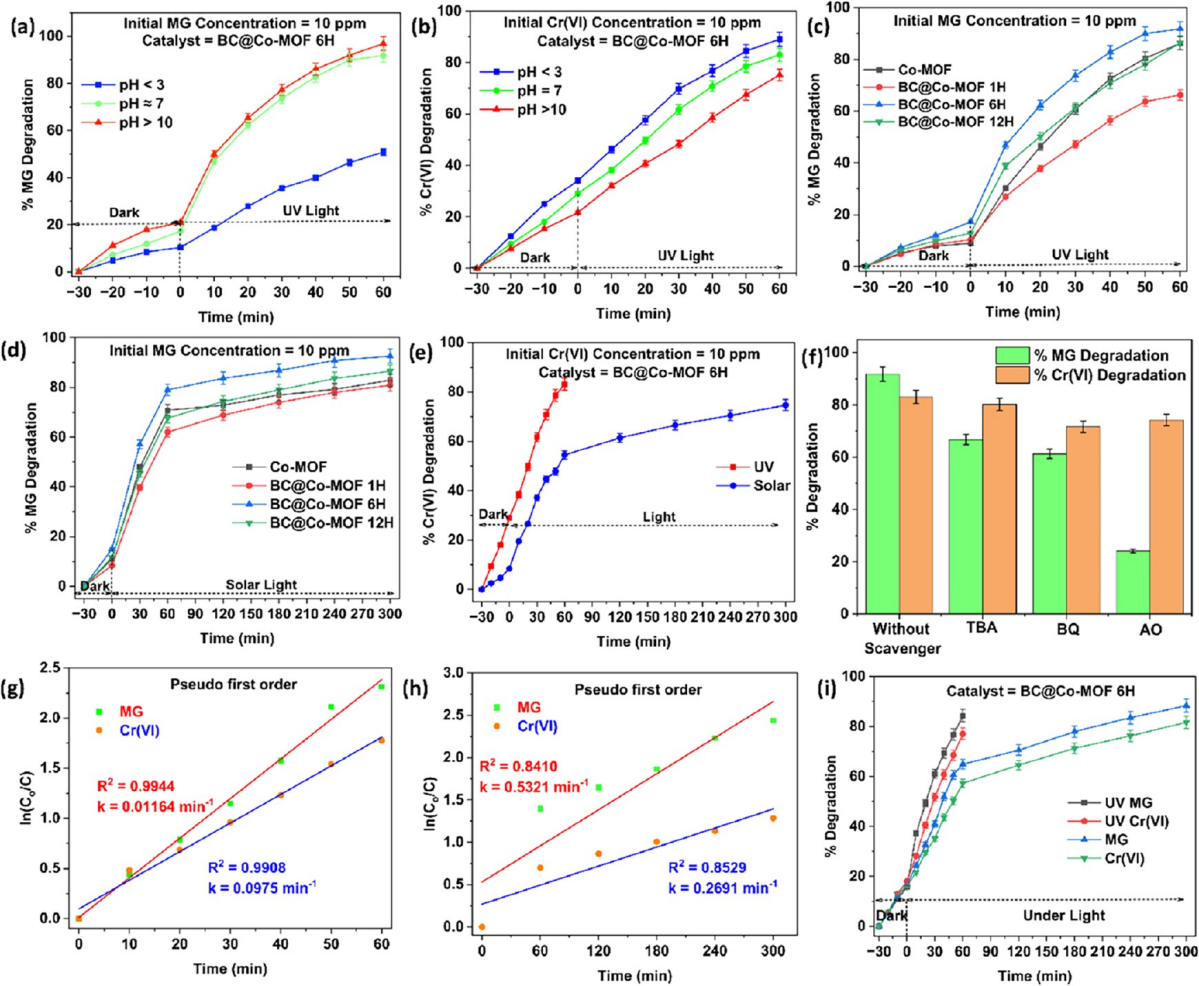
(a) Effect of pH on photocatalytic degradation of MG under UV light, (b) effect of pH on photocatalytic degradation of Cr­(VI) under UV light, (c) photocatalytic degradation of MG under UV light irradiation, (d) photocatalytic degradation of MG under solar light irradiation, (e) photocatalytic degradation of Cr­(VI) under UV and solar light, (f) radical scavenger studies, (g) rate kinetics under UV light, (h) rate kinetics under solar light, and (i) photocatalytic degradation of a mixture of MG and Cr­(VI)­using BC@Co-MOF 6H in the presence of UV and solar light.

Arulmurugan et al. studied the photocatalytic efficiency of WO_3_/graphene composites for the photocatalytic degradation of MG in the pH range of 1–11. The results showed maximum degradation of MG (95.2%) at neutral pH (pH = 7). The point of zero charge (PZC) of the WO_3_/graphene composite in the above study was observed between pH 5 and pH = 7. At the solution pH values below the PZC, the catalyst surface was positively charged, reducing adsorption and degradation efficiency. At pH values above the PZC, the catalyst surface was negatively charged, enhancing electrostatic attraction of cationic MG molecules, boosting their adsorption and degradation. However, extremely basic conditions lead to excessive OH^–^ ion formation, reducing the overall efficiency.^43^ Similarly, in another study by Chen et al., they observed that the degradation efficiency of MG increased from 88% at acidic pH to 98.91% at basic pH using Bi_2_O_3_–TiO_2_/powdered activated carbon composite catalyst. The increase in degradation efficiency was attributed to enhanced generation of ROS and improved adsorption of cationic MG dye on the negatively charged catalyst surface.^44^

#### 3.2.3 Effect of Irradiation Source

The photocatalytic activity of Co-MOF and its composites was evaluated under UV and solar light to assess the effect of the irradiation source. The reactions were carried out for the degradation of MG and Cr­(VI) at optimal conditions (initial concentration = 10 ppm and pH ≈ 7). In both cases, an adsorption–desorption equilibrium was evaluated by keeping the reaction mixture in the presence of the desired catalyst in the dark for 30 min. A minimal degradation was observed in dark conditions for both MG and Cr­(VI). Among the synthesized materials, BC@Co-MOF 6H showed the highest photocatalytic degradation of MG in the presence of UV (Figure 9c) as well as solar light (Figure 9d). Using BC@Co-MOF 6H, the % MG degradation after 60 min in the presence of UV and solar light was 92.5 and 79%, respectively. Using the same catalyst, about 90% MG degradation was observed in the presence of solar light after a reaction time of 300 min. The result indicates the superior performance of the synthesized composite, BC@Co-MOF 6H, under UV as well as visible light for the degradation of MG. The effect of various light sources on the photocatalytic degradation of Cr­(VI) using BC@Co-MOF 6H is shown in Figure 9e. The % Cr­(VI) degradation under UV light and solar light was 83.06% (after 60 min) and 74.66% (after 300 min), respectively. The results reveal that, using BC@Co-MOF 6H, photocatalytic degradation of Cr­(VI) is more efficient in the presence of UV light than solar light. The superior photoactivity of BC@Co-MOF 6H under solar irradiation offers various advantages such as cost-effectiveness, sustainability, and environmental friendliness. Further, the results indicate the suitability of BC@Co-MOF 6H as a green photocatalyst for large-scale wastewater treatment.

#### 3.2.4 Effect of Scavengers

A photocatalytic degradation mechanism mainly involves three species: superoxide radicals (^•^O_2_^–^), hydroxyl radicals (^•^OH), and holes (h^+^). In this study, the mechanism of degradation was investigated by adding different radical scavengers, namely, tert-butanol (TBA), benzoquinone (BQ), and (AO), to scavenge ^•^OH, ^•^O_2_^–^, and h^+^, respectively. Figure 9f shows the degradation of MG dye and Cr­(VI) using BC@Co-MOF 6H with and without a scavenger. The above reactions were carried out at a neutral pH of the solution, at an initial MG concentration of 10 ppm, and in the presence of UV light. The presence of radical scavengers showed a negative effect on the degradation of MG. The % MG degradation in the presence of TBA, BQ, and AO was 66.78, 61.28, and 24.06%, respectively. Among the radical scavengers, AO showed maximum negative effect on the % MG degradation, suggesting h^+^ as the primary radical species for the degradation of MG.^45,46^ On the other hand, the % Cr­(VI) degradation in the absence of any scavenger was 83.06%. The % Cr­(VI) degradation in the presence of TBA, BQ, and AO was reduced to 80, 71.65, and 74.23%, respectively. The results indicate dominance of ^•^O_2_^–^, and h^+^ radicals for the degradation of Cr­(VI). The results were consistent with the earlier studies.^47^

### 3.3 Reaction Kinetics

The experimental results for the photocatalytic degradation of MG dye and Cr­(VI) using BC@Co-MOF 6H at the optimum reaction conditions (at a neutral pH of the solution, at an initial concentration of 10 ppm) under different light sources were fitted using a pseudo-first-order kinetic model (Figure 9g,h). From Figure 9g, a strong linear relationship (*R* ^2^ > 0.99) between time and ln­(*C*_0_/*C*) was observed for MG as well as for Cr­(VI). This suggests that photocatalytic degradation of both MG and Cr­(VI) in the presence of UV light followed pseudo-first-order kinetics. The apparent rate constants (*k*) for the UV light-mediated photocatalytic degradation of MG and Cr­(VI) were 0.01164 and 0.0975 min^–1^, respectively. On the other hand, the apparent rate constants for photocatalytic degradation of MG and Cr­(VI) under solar light (Figure 9h) were 0.5321 and 0.2691 min^–1^, respectively.

### 3.4 Photocatalytic Degradation Study on a Mixture of MG and Cr­(VI)

The photocatalytic activity of the BC@Co-MOF 6H composite was also studied for the degradation of a solution containing a mixture of MG and Cr­(VI). At the optimum conditions (at a neutral pH of the solution, at an initial concentration of 10 ppm), the reactions were conducted for 60 and 300 min in the presence of UV light and solar light, respectively. The results of the above experiments are shown in Figure 9i. Under UV irradiation, there was 84.31% MG degradation and 77.09% Cr­(VI) degradation. On the other hand, under solar light, there was 88.36% MG degradation and 81.65% Cr­(VI) degradation. The results demonstrate the high photocatalytic activity of BC@Co-MOF 6H even in the presence of mixed pollutants. Tables 2 and 3 provide a comparison of the results obtained in this study with the earlier published literature on the photocatalytic degradation of MG and Cr­(VI), respectively.

**Table 2:** Photocatalytic Degradation of MG Using Various Photocatalysts

sr. no.	photocatalyst	operating parameters	time of reaction (min)	% degradation and rate constant	references
1	ZnO	amount of the catalyst = 50 mg/L, initial concentration of MG = 100 ppm, light source = UV light (λ = 254 nm)	180	99.8%, *k* = 0.81 min^–1^	^48^
2	Ni-Co/TiO_2_	amount of the catalyst = 1 g/L, initial concentration of MG = 10 ppm, light source = UV light	60	82.39%, *k* = 0.0119 min^–1^	^19^
3	g-C_3_N_4_-Ag-TiO_2_	amount of the catalyst = 1 g/L, initial concentration of MG = 10 ppm, light source = 250 W xenon lamp	60	65.5%, *k* = 0.022 min^–1^	^49^
4	LaCeO_3_/CuO	amount of the catalyst = 30 mg, initial concentration of MG = 5.0 ×10^–6^ M, light source = 50 W LED lamp	120	92.88%, *k* = 6.19 × 10^–2^ min^–1^	^50^
5	Cu-based MOF	amount of the catalyst = 1.25 g/L, initial concentration of MG = 1 ppm, light source = UV–visible light	130	97.61%	^51^
6	Ni-based MOF	amount of the catalyst = 1.25 g/L, initial concentration of MG = 1 ppm, light source = UV–visible light	130	84.76%	^51^
7	La-BDC MOF	amount of the catalyst = 50 mg/100 mL, initial concentration of MG = 20 ppm, light source = UV light	60	97%, *k* = 0.0637 min^–1^	^52^
8	Cu_3_Mo_2_O_9_@MWCNT	amount of the catalyst = 20 mg, initial concentration of MG = 50 mL of 5 × 10^–5^ MG solution, light source = 50 W LED bulb	180	96%, *k* = 0.02114 min^–1^	^53^
9	BC@Co-MOF 6H	Amount of the catalyst = 50 mg/100 mL, initial concentration of MG = 10 ppm, light source = UV light (125 W high-pressure mercury vapor lamp)	60	92.52%, *k* = 0.01164 min^–1^	this study
10	BC@Co-MOF 6H	amount of the catalyst = 50 mg/100 mL, initial concentration of MG = 10 ppm, light source = natural sunlight	300	91.8%, *k* = 0.5321 min^–1^	this study

**Table 3:** Photocatalytic Degradation of Cr­(VI) Using Various Photocatalysts

sr. no.	photocatalyst	operating parameters	time of reaction (min)	% degradation	references
1	HSB@TiO_2_	amount of the catalyst = 100 mg, initial concentration of Cr(VI) = 30 ppm, light source = metal-halide lamp 300 W	210	48.5%	^54^
2	g-C_3_N_4_/Bi_2_Ti_2_O_7_/TiO_2_	amount of the catalyst = 100 mg, concentration = 20 ppm, light source = 250 W xenon lamp	120	95%, *k* = 0.0242 min^–1^	^55^
3	Bi_2_S_3_/BiVO_4_/TiO_2_	amount of the catalyst = 2 × 5 cm^2^, initial concentration of Cr(VI)= 5 ppm, light source = solar Xe lamp system with AM1.5G filter	100	96.1%, *k* = 0.0336 min^–1^	^56^
4	ZnO/ZrO_2_	initial concentration of Cr(VI)= 1 mM, light source = mercury vapor lamp – 250 W	150	63%, *k* = 6.19 × 10^–2^ min^–1^	^57^
5	UiO-66-NH2@HDU-25	amount of the catalyst = 0.2 mg/mL, initial concentration of Cr(VI)= 15 ppm, light source = 300 W xenon lamp	30	99.1%, *k* = 0.00457 min^–1^	^51^
6	Zn-AgIn5S8/CdS/SrGO	amount of the catalyst = 1 g/L, initial concentration of Cr(VI)= 50 ppm, light source = solar light	180	85%	^58^
7	BOC/BTO@Co-MOF	amount of the catalyst = 0.5 mg/mL, initial concentration of Cr(VI)= 30 ppm, light source = 300 W xenon lamp	60–90	96.5%, *k* = 0.0637 min^–1^	^59^
8	BC@Co-MOF 6H	amount of the catalyst = 50 mg/100 mL, initial concentration of Cr(VI)= 10 ppm, light source = UV (125 W high-pressure mercury vapor lamp)	60	83.06%, *k* = 0.0975 min^–1^	this study
9	BC@Co-MOF 6H	amount of the catalyst = 50 mg/100 mL, initial concentration of Cr(VI)= 10 ppm, sunlight	300	74.66%, *k* = 0.2691 min^–1^	this study

### 3.5 Photocatalytic Degradation Mechanism

The intermediate products of the photocatalytic degradation of MG were identified using LC-MS, and based on that, a plausible degradation mechanism is proposed in Figure 10. The degradation experiments were carried out for 60 min under UV light at the optimum reaction conditions in the presence of BC@Co-MOF 6H, and the aliquots were withdrawn at an interval of 10 min. The mass spectra of MG are provided in Figure S3 illustrating progressive breakdown with reaction time. After 60 min of reaction, the peak of MG corresponding to the *m*/*z* = 329 completely disappeared, and several new peaks at *m*/*z* = 274, *m*/*z* = 212, *m*/*z* = 122, *m*/*z* = 90, and *m*/*z* = 74 appeared in the mass spectra of the degraded sample. The complex structure of the dye was initially broken down by demethylation. As the reaction progressed, the intermediates were further broken down by hydroxylation reaction into simpler compounds.^60,61^ The photocatalytic Cr­(VI) reduction mechanism can be explained using the equation mentioned below. In the CB, photoelectrons attack Cr­(VI) and ultimately convert it to Cr­(III), whereas holes at the VB react with water to produce protons and oxygen, as indicated by the equation.

3 $$\mathrm{Cr}\left(\mathrm{VI}\right)+e^{-}\to \mathrm{Cr}\left(\mathrm{IV}\right)+e^{-}\left(\mathrm{CB}\right)\to \mathrm{Cr}\left(\mathrm{III}\right)$$

4 $$2H_{2}O+4h^{+}\left(\mathrm{VB}\right)\to O_{2}+4H^{+}$$

**Figure 10:**
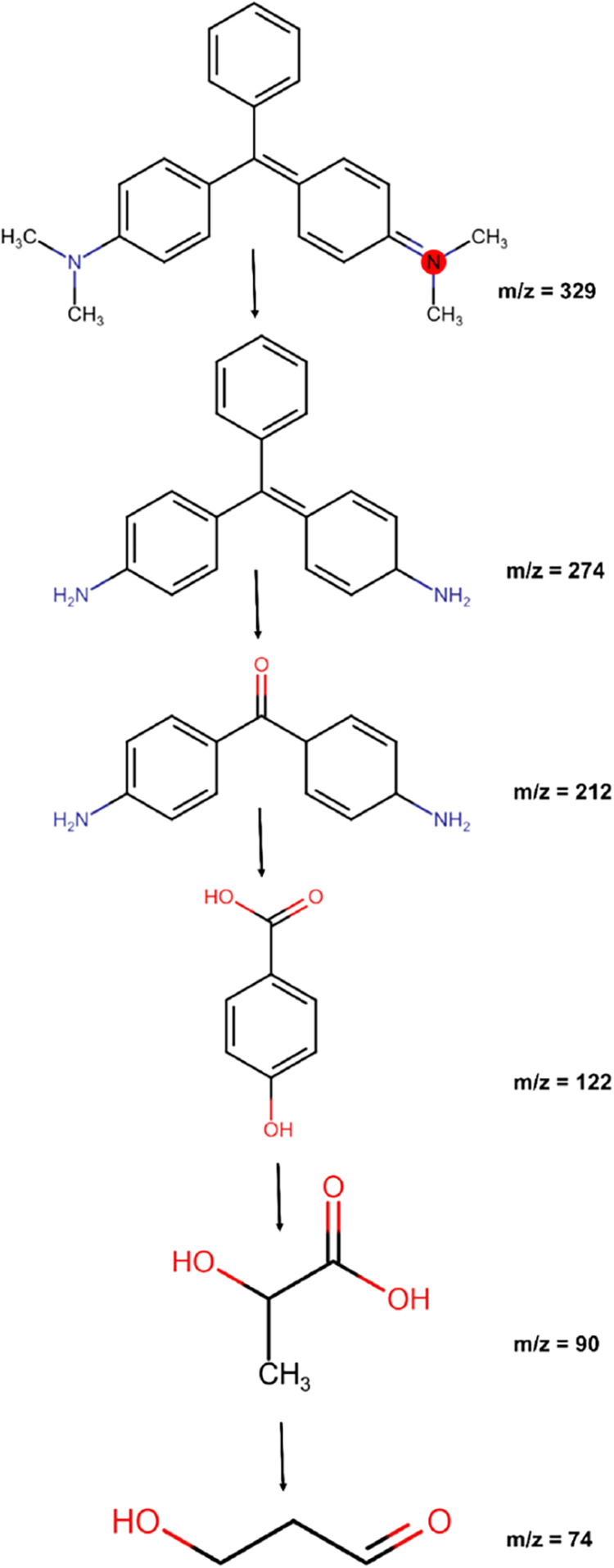
Proposed pathway for the photocatalytic degradation of MG dye using BC@Co-MOF 6H.

### 3.6 Recovery and Recyclability Studies

The reusability of the BC@Co-MOF 6H composite was tested for four successive cycles for the photocatalytic degradation of MG under UV irradiation (Figure 11a). The photocatalytic activity of the catalyst gradually decreased after each cycle, which was evident from the decrease in the % MG degradation in the subsequent cycles. The % MG degradation was 92.58, 81.62, 75.09, and 67.61% for the first, second, third, and fourth cycles. The reduction in the photocatalytic activity of the catalyst may be attributed to the loss of surface active sites. To confirm this assumption, the spent catalyst recovered after 4 reaction cycles was characterized using XPS, XRD, and EDS spectra. The obtained results were compared with those of the fresh catalysts. The XPS spectra (Figure 11b) of the spent catalyst revealed a reduction in the Co 2p peak. Similarly, the XRD pattern of the spent catalyst (Figure 11c) showed a reduction in the intensity of the characteristic diffraction peaks. The elemental composition of the catalyst, with the help of EDS showed the presence of 23 wt % Co (Figure 11d) which was lower by about 6.5 wt % compared to the fresh catalyst (Figure 2c). The comparison of the results for the spent and fresh catalyst indicates a loss of Co-MOF active sites from the surface of BC.

**Figure 11:**
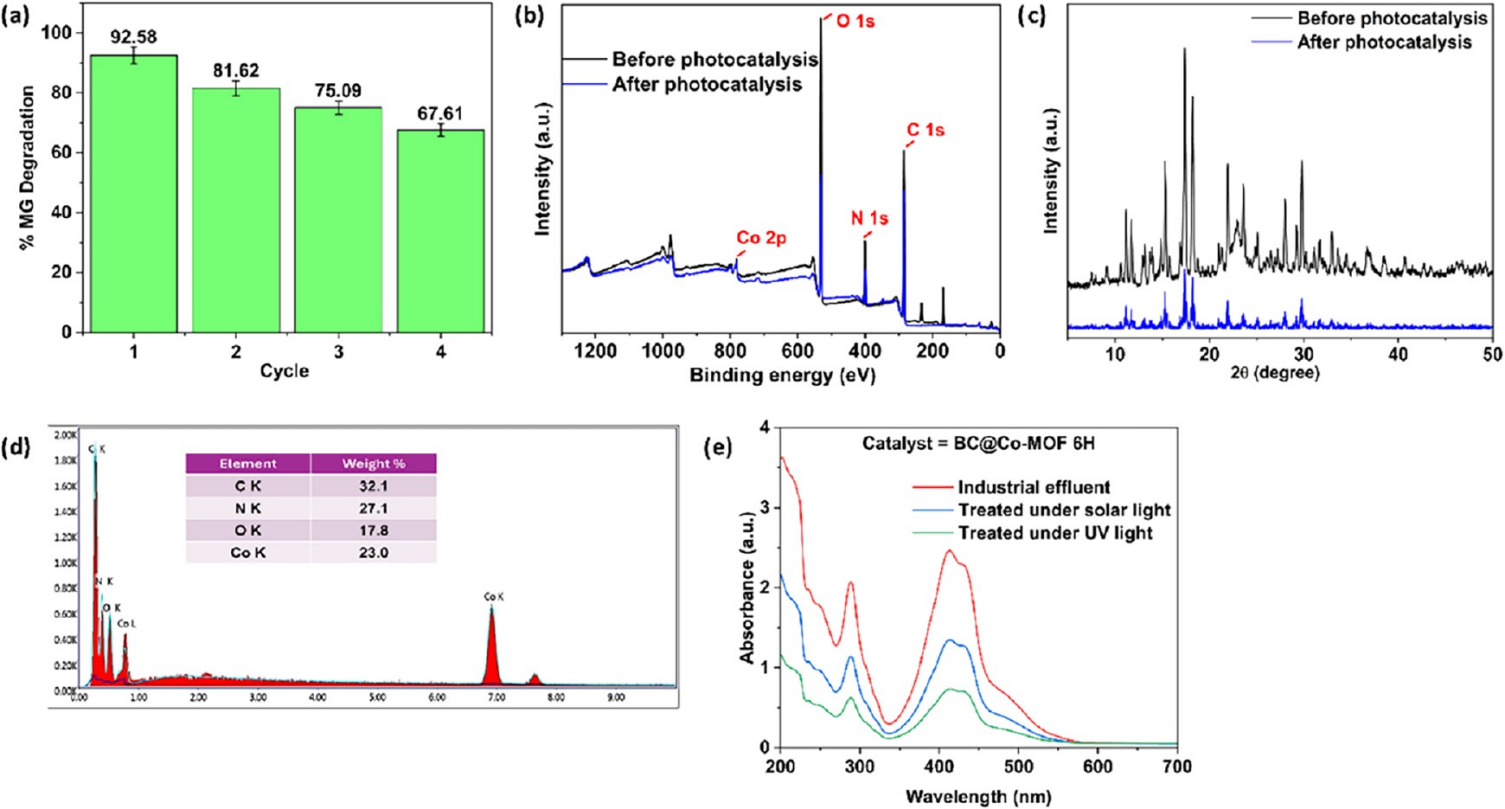
(a) Recyclability study of BC@Co-MOF 6H for the photocatalytic degradation of MG, (b) XPS of the spent and fresh BC@Co-MOF 6H, (c) XRD of the spent and fresh BC@Co-MOF 6H, (d) EDS spectra of the spent BC@Co-MOF, and (e) UV–vis absorption spectra of the treated and untreated pharmaceutical effluent.

### 3.7 Photocatalytic Degradation of Real-Time Pharmaceutical Effluent Using BC@Co-MOF 6H

To investigate the potential of BC@Co-MOF 6H from a practical point of view, photocatalytic degradation of a real-time pharmaceutical effluent was carried out. In the above experiment, 50 mg of BC@Co-MOF 6H was added to a 100 mL effluent sample, and the reaction mixture was subjected to photocatalytic degradation under UV light and solar light. The absorbance spectrum of the effluent sample before and after the degradation was recorded and compared. Figure 11e shows the UV–vis absorbance spectra of untreated effluent, effluent treated using UV light, and effluent treated using solar light. The absorbance of the treated effluent was less compared to the untreated solution, indicating degradation of various pharmaceutical pollutants from the effluent. The absorbance of the effluent treated under UV light was approximately 4-fold lower compared to the untreated solution. On the other hand, there was about a 2-fold reduction in the absorbance for the effluent treated under solar light. These results indicate an effective degradation of the real-time effluent, and thereby a strong potential of the reported material for large-scale applications.

### 3.8 Phytotoxicity Studies

Phytotoxicity studies were performed to study the safety of the treated water post photocatalytic degradation prior to its release into the environment. For this, seed germination and seedling growth were examined after exposure to DI water (control), an untreated MG solution, and various photocatalytically treated MG solutions. The results of the phytotoxicity studies on Mung beans and chickpea are shown in Figures 12 and 13, respectively. The results indicate a strong inhibition of seed germination in the presence of untreated and BC-treated MG solutions. On the other hand, the seeds in the presence of photocatalytically degraded solutions using Co-MOF or BC@Co-MOF 6H showed notable growth in the shoots. In the Co-MOF or BC@Co-MOF 6H-treated solutions, the Mung bean seeds began germination from day 1 itself, with an average shoot length of 0.56 cm, which gradually increased to 1.86 cm at the end of day 4. The shoot length for Chickpea seeds at the end of 4 days was 0.19 and 0.3 cm when exposed to Co-MOF or BC@Co-MOF 6H-treated solutions, respectively. The detailed observation for the variation in the shoot lengths of Mung beans and Chickpea seeds exposed to different solutions is presented in Table 4. The % growth inhibition calculated using eq 2 for mung beans and chickpea seeds exposed to BC@Co-MOF 6H-treated solution was 30.07 and 44.4%, respectively.

**Figure 12:**
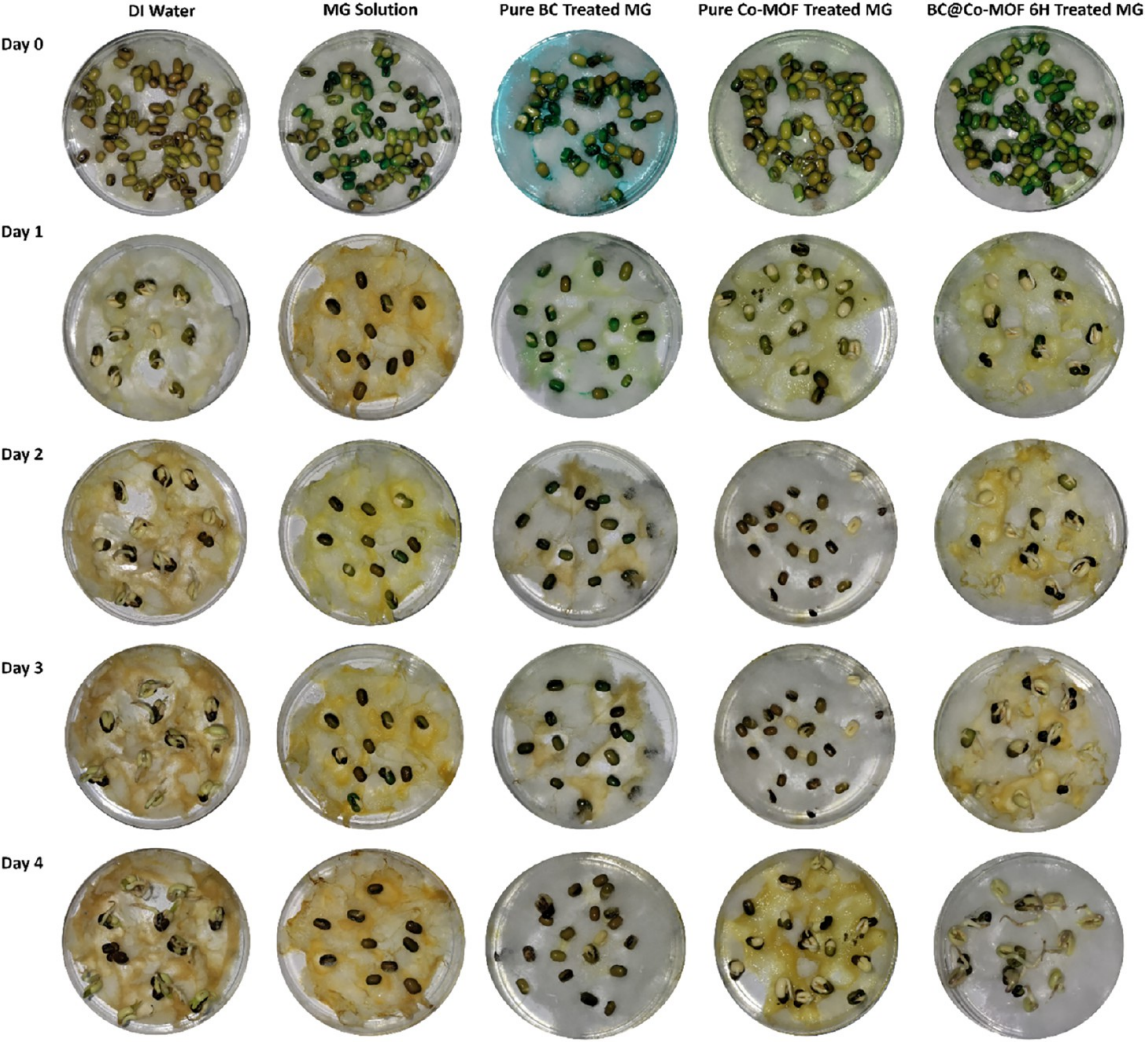
Phytotoxicity study on mung bean (*Vigna radiata* L.).

**Figure 13:**
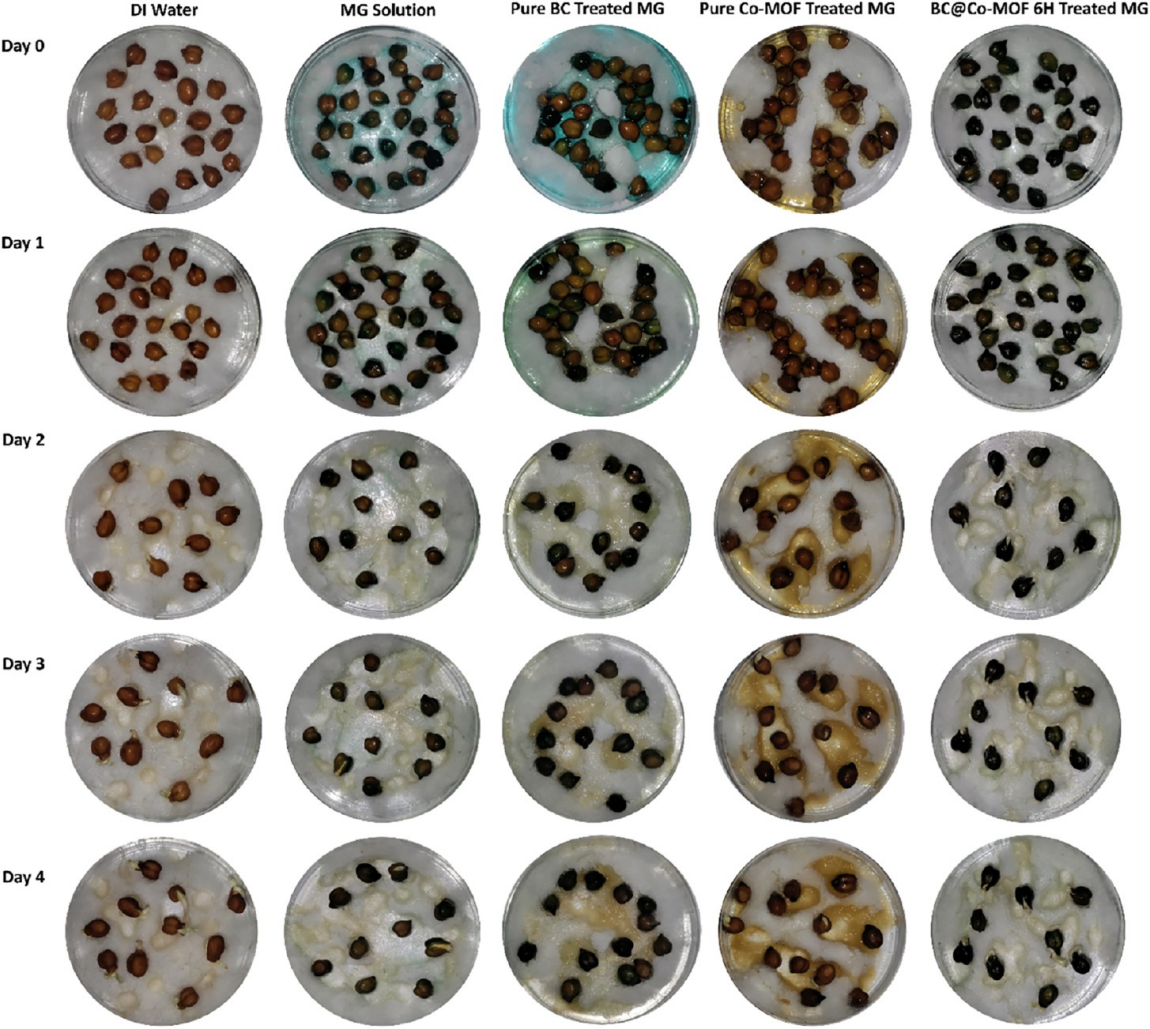
Phytotoxicity study on chickpea (*Cicer arietinum* L.).

**Table 4:** Phytotoxicity Assessment of Untreated and Treated MG Solution Using Mung Bean (*Vigna radiata* L.) and Chickpea (*Cicer arietinum* L.) Seeds

		seed length (cm)				
				photocatalytic degradation		
seed type	day	DI water	10 ppm MG solution	BC-treated MG	Co-MOF-treated MG	BC@Co-MOF 6H-treated MG
Mung bean (*Vigna radiata* L.)	day 1	0.95	no germination	no germination	0.23	0.56
	day 2	1.45	no germination	no germination	0.70	1.37
	day 3	2.19	no germination	no germination	1.14	1.62
	day 4	2.66	no germination	no germination	1.20	1.86
Chickpea (*Cicer arietinum* L.)	day 1	no germination	no germination	no germination	no germination	no germination
	day 2	no germination	no germination	no germination	no germination	no germination
	day 3	0.35	no germination	no germination	0.10	0.21
	day 4	0.54	no germination	no germination	0.19	0.30

The complete inhibition of seed germination in the untreated MG solution highlights its acute phytotoxicity, rendering it unsuitable for discharge into agricultural or aquatic environments. In contrast, photocatalytic treatment effectively decolorized and detoxified the dye, resulting in a significant reduction in toxicity.^62,63^ The partial yet definitive restoration of seed germination and seedling growth indicates that the treated effluents are either nontoxic or substantially less hazardous than the untreated ones.

## 4 Conclusions

The present study shows a significant degradation of MG and Cr­(VI) using BC@MOF biocomposite as an efficient photocatalyst. The BC@Co-MOF was successfully synthesized using the *in situ* precipitation technique. The incorporation of Co-MOF on the BC matrix improved the photocatalytic performance by enhancing the active sites and improving mass transfer, uniform dispersion, light absorption, and charge carrier separation. Initially, Co-MOF exhibited a flake-like crystalline structure. When loaded onto the BC matrix and immersed in the precursor solution for 1, 6, and 12 h, its morphology evolved, forming rod-like structures at early stages and progressively overgrowing at longer immersion times. Among the synthesized samples, BC@Co-MOF 6H exhibited the highest photocatalytic activity.

The effect of initial MG concentration, pH, and source of irradiation on photocatalytic degradation in the presence of BC@Co-MOF 6H was studied. A maximum 92.52% degradation of MG was observed with an initial concentration of 10 ppm at a neutral pH of the solution and under UV light. At similar conditions, the % Cr­(VI) degradation was found to be 71.67%. The photocatalytic degradation of MG and Cr­(VI) followed pseudo-first-order kinetics under UV and solar light. The effect of scavengers revealed the dominance of h^+^ radicals for the degradation of MG and ^•^O_2_^–^, and h^+^ for the degradation of Cr­(VI). A possible degradation mechanism was proposed with the help of LC-MS analysis. The recyclability studies demonstrated superior photocatalytic activity of BC@Co-MOF 6H over 4 successive cycles. The BC@Co-MOF 6H exhibited superior activity for the photocatalytic degradation of a real-time pharmaceutical effluent in the presence of UV as well as solar light. The phytotoxicity studies on the germination of mung bean (*Vigna radiata* L.) and chickpea (*Cicer arietinum* L.) showed constant shoot growth over 4 days, indicating the suitability of treated water for safe disposal and environmental use. This work paves a path for expanding the utilization of the BC based composite catalyst to a wider range of pollutant degradation, thereby contributing to the development of eco-friendly wastewater treatment technologies.

## Supplementary Material



## Notes

Table S1: Various treatment methods for the removal of MG dye. Table S2: Various treatment methods for the removal of Cr­(VI). Figure S1: FESEM image of BC matrix. Figure S2: XPS spectra of Co-MOF and its composites. Figure S3: Mass Spectra of MG obtained from photocatalytic degradation studies using BC@ Co-MOF 6H at the interval of 0, 10, 20, 30, 40, and 60 min. (PDF)
