# Supplementary material for: Efficient Photocatalytic Degradation of Malachite Green and Cr(VI) Using Co-MOF and Bacterial Cellulose@Co-MOF Biocomposite: A Green Approach
Source: ACS Omega. 2025 Sep 23;10(39):45965–81. doi: 10.1021/acsomega.5c06750 (PMC12509024; doi:10.1021/acsomega.5c06750)
Supplement: Supplementary file 1 [file ao5c06750_si_001.pdf]

Supporting Information

Efficient Photocatalytic Degradation of Malachite Green and Cr (VI) using Co-MOF and Bacterial Cellulose@Co-MOF Bio Composite: A Green Approach

Krushika Mhalshekar[1], Subhesh Selvam[2], Aditya Sahoo[1], Mani Pujitha Illa[2], Mrunalini Gaydhane[1] and Sharad Sontakke[1]*

[1] Nano Prakruti Research Lab, Department of Chemical Engineering, Birla Institute of Technology and Science, Pilani, K K Birla Goa Campus, Goa, 403726, India

[2] Battery Materials Lab, Department of Metallurgical and Materials Engineering, National Institute of Technology Tiruchirappalli, Tiruchirappalli – 620015, Tamil Nadu, India

* Corresponding author's e-mail-id: mrunalinig@goa.bits-pilani.ac.in

Table S1. Various treatment methods for the removal of MG dye

Method	MG dye				Reference
	Material	Reaction condition	Reaction Time	% Removal	
Adsorption	$Zn_{1-x}Cu_xO$	adsorbent dosage = 1 g/l, conc = 5 ppm	120 min	95.2%	1
Oxidation	Ag-Pt/$K_2S_2O_8$	Catalyst dosage = 0.3 g/*L*, Conc= 1.25 mM, *T* = 25 °C, pH = 7.0	50 min	94.2%	2
Photocatalysis	$LaCeO_3$/CuO	Catalyst = 30 mg, Conc	120 min	92.88%	3

		= 5.0×10^{-6} M, 50 W LED lamp			
	Cu-based MOF	Catalyst = 1.25 g/L, Conc = 1 PPM, UV–visible light	130 min	97.61%	[4]

Table S2. Various treatment methods for the removal of Cr(VI)

Method	Cr(VI)				Reference
	Material	Reaction condition	Reaction Time	Results	
Adsorption	PAN-GO-Fe3O4	Adsorbent dose = 0.6 g/L, Conc =50 mg/L,	70 min	124.37 mg/g	[5]
	Fe-modified rice-straw biochar	adsorbent dosage = 2.67 g/L, pH 2.5, temperature 25 ± 2 ˚C	60-120 min	98%	[6]
Photocatalysis	ZnO/ZrO_2	Concentration = 1 mM, mercury vapour lamp −250 W	150 min	63%	[7]Sathisha
	BOC/BTO@Co-MOF	Catalyst = 0.5 mg/ mL, Concentration = 30 PPM, 300W Xenon lamp	60-90 min	96.5%	[8]

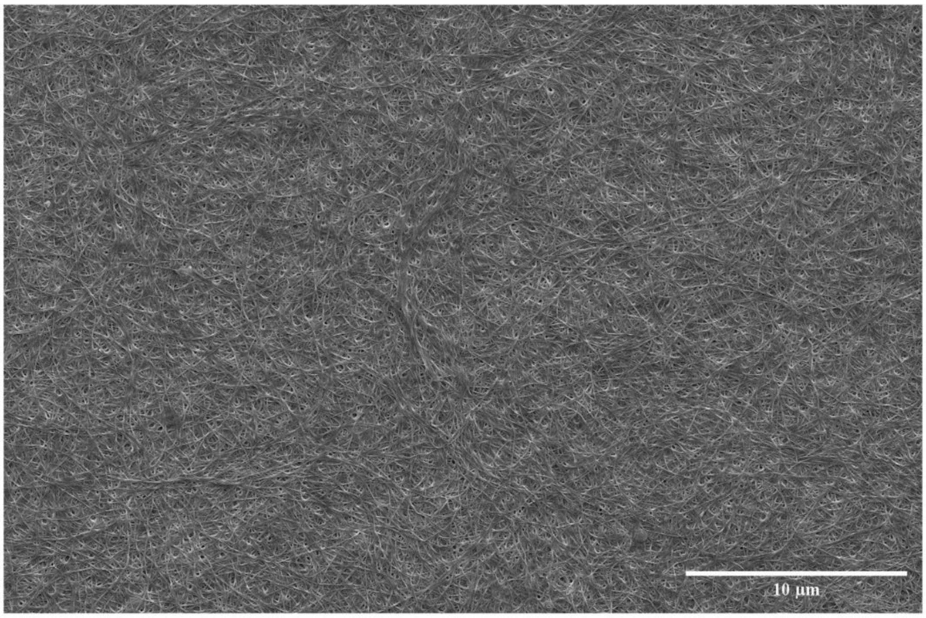

Figure S1. FESEM image of BC matrix.

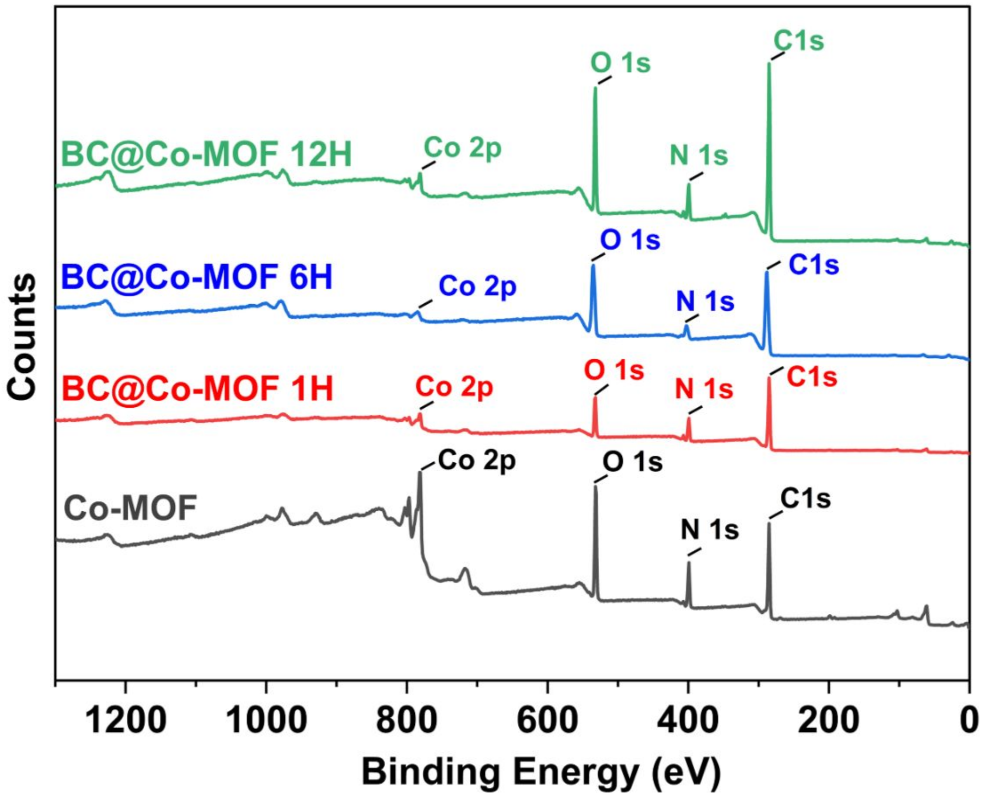

Figure S2. XPS spectra of Co-MOF and its composites.

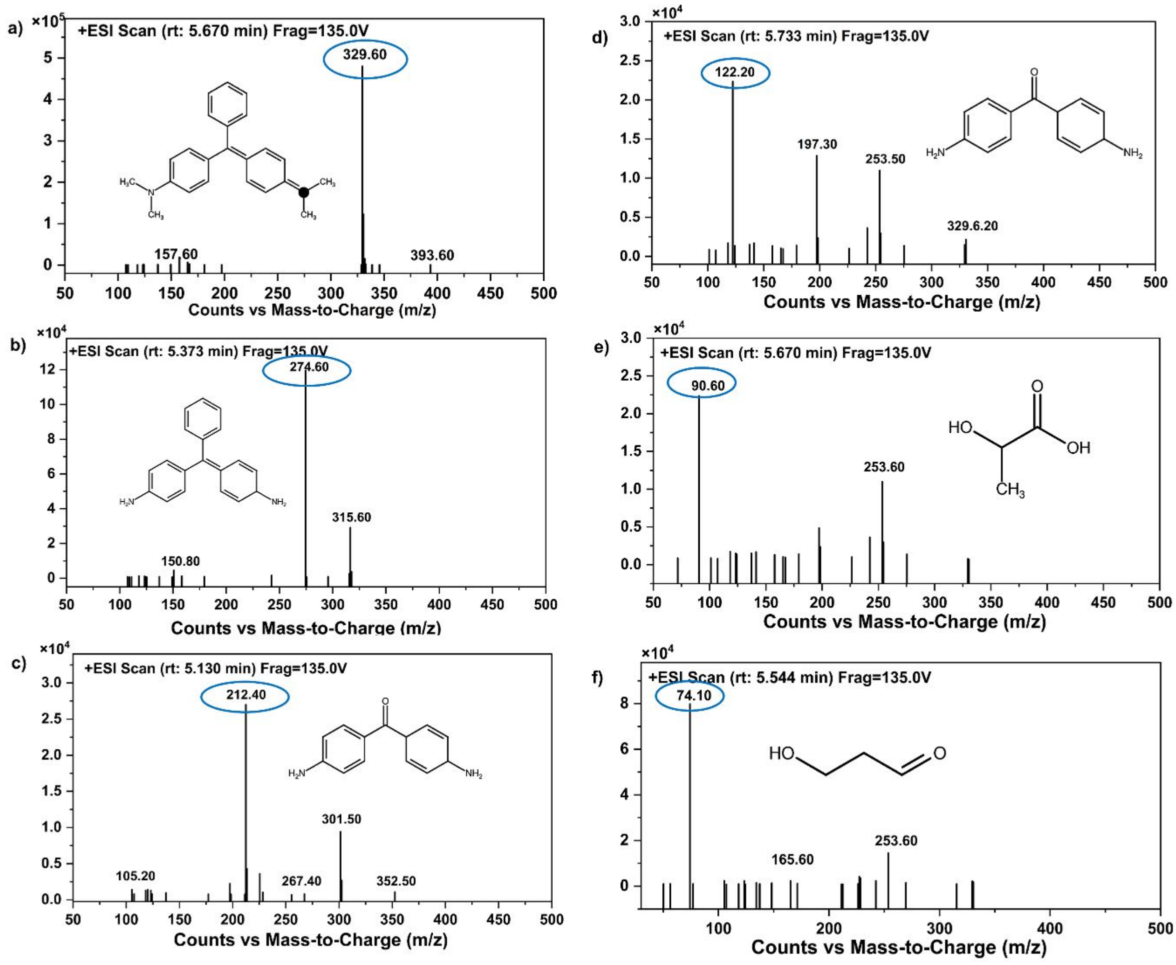

Figure S3: Mass Spectra of MG obtained from photocatalytic degradation studies using BC@ Co-MOF 6H at the interval of a) 0 min, b) 10 min, c) 20 min, d) 30 min, e) 40 min and f) 60 min

References

(1) Tekyeh, M. N.; Mehrparvar, D.; Moradian, R.; Mahdavi, S. Surface Adsorption of Malachite Green Dye from Aqueous Solution Using Novel Synthesized Adsorbent ZnO, Zn1-XFexO and Zn1-XCuxO Nanoparticles. *Desalination Water Treat* **2025**, *322*, 101202.

(2) Mohammed Al-Balawi, A.; Zaheer, Z.; Kosa, S. A. Silver-Platinum Bimetallic Nanoparticles as Heterogeneous Persulfate Activator for the Oxidation of Malachite Green. *Arabian Journal of Chemistry* **2023**, *16* (8), 104863.

(3) Irbati, R. D. F.; Apriandanu, D. O. B.; Rahayu, M.; Ananda, V. R.; Yusuf, M. R.; Chandren, S.; Yulizar, Y. Green Fabrication of Novel $LaCeO_3$ Decorated with CuO Using Kigelia Africana (Lamb) Benth Leaf Extract for Photocatalytic Degradation of Malachite Green. *Nano-Structures and Nano-Objects* **2025**, *42*, 101474.

(4) Betseba, A. H. H.; Shaji, Y. C. Surface Area Enhanced Synthesis of Cu and Ni-Based Metal-Organic Frameworks for Photocatalytic Degradation of Malachite Green Dye and Anticancer Drug Delivery Applications. *J Drug Deliv Sci Technol* 2025, *111*, 107138.

(5) Sahoo, S. K.; Panigrahi, G. K.; Sahoo, J. K.; Pradhan, A. K.; Purohit, A. K.; Dhal, J. P. Electrospun Magnetic Polyacrylonitrile-GO Hybrid Nanofibers for Removing Cr(VI) from Water. *J Mol Liq* 2021, *326*, 115364.

(6) Yu, C.; Yang, J. Removal of Cr(vi) in Wastewater by Fe-Mn Oxide Loaded Sludge Biochar. *RSC Adv* 2024, *14* (17), 11746–11757.

(7) Sathisha, H. C.; Anitha; Krishnamurthy, G.; Nagaraju, G. Facile Green Synthesis of ZnO/ZrO2 Nanocomposite for Photocatalytic Degradation and Chromium (VI) Reduction. *J Cryst Growth* 2025, *651*, 128009.

(8) Sompalli, N. K.; Li, Y.; Li, J.; Kuppusamy, S. An Innovative Triple Interface Reinforced Photocatalytic System Based on BiOCl/BaTiO3@Co-BDC-MOF Composite for the Simultaneous Detoxification of Cr(VI) and Sulfamethoxazole. *Environ Res* 2024, *259*, 119532.
